# Sustaining the gains made in malaria control and elimination

**DOI:** 10.1186/s40249-015-0057-x

**Published:** 2015-05-03

**Authors:** Randall A Kramer, Adriane Lesser

**Affiliations:** Duke Global Health Institute and Nicholas School of the Environment, Duke University, Durham, NC USA; Duke Global Health Institute, Duke University, Durham, NC USA

**Keywords:** Malaria control, Elimination, Resistance, Inter-sectoral

## Abstract

Significant progress has been made in the last 25 years to reduce the malaria burden, but considerable challenges remain. These gains have resulted from large investments in a range of control measures targeting malaria. Fana and co-authors find a strong relationship between education level and net usage with malaria parasitemia in pregnant women, suggesting the need for targeted control strategies. Mayala and co-workers find important links between agriculture and malaria with implications for inter-sectoral collaboration for malaria control.

**Electronic supplementary material:**

The online version of this article (doi:10.1186/s40249-015-0057-x) contains supplementary material, which is available to authorized users.

## Multilingual abstracts

Please see Additional file 1 for translations of the abstract into the six official working languages of the United Nations.

## Background

Since 2000, there has been a huge ramp up of international funding to fight malaria and the latest statistics show the resulting progress -- a 46% relative decrease in malaria infection prevalence in sub-Saharan Africa among children 2–10 years old between 2000 and 2013 and an estimated 4.3 million deaths averted globally from 2001 to 2013 [1]. These gains reflect significant investments in a range of control measures targeting malaria, including both mosquito vector control and disease management strategies [2]. These successful investments have led to calls for malaria elimination in some countries that have had highly successful control efforts [1,3]. Despite these significant gains, malaria remains a major global health concern. In 2013, there were an estimated 198 million malaria cases worldwide and 584,000 deaths were attributable to malaria [1]. Although international and domestic funding to control malaria totaled $2.7 billion in 2013, this is estimated to be about half of what is needed to reach global targets for reducing the burden of malaria [1].

Two major concerns regarding further malaria reductions are insecticide resistance and drug resistance. Mosquitos have developed resistance to at least one insecticide in 53 out of 65 countries providing data over the period 2010–2013, meaning that insecticides used to treat nets and spray houses may be gradually losing their effectiveness. The second concern is resistance of malaria to the primary drug for treating malaria cases, artemisinin-based combination therapies (ACT), which as a result may not be as effective in the long run. Resistance to ACT by *P. falciparum*, the most deadly of the malaria parasites, has been suspected or confirmed in 4 Southeast Asian countries [4].

Therefore, to sustain and expand the gains made to-date, and to move towards elimination, there is a need to more fully understand how best to expand and refine existing measures as well as develop novel approaches to control.

## Discussion

Two articles in this issue examine malaria management strategies in the context of a rapidly changing malaria control environment. Fana et al. [5] study malaria prevalence among pregnant women in Northwest Nigeria. Using data from interviews and microscopy of blood smears among a semi-urban population, they are able to compare several factors with malaria prevalence. They find high malaria prevalence (41.6% overall), much higher than in many other parts of sub-Saharan Africa. Malaria prevalence is particularly high among those pregnant women with no schooling and who do not use nets. The strong associations between both education level and ITN usage with malaria parasitemia point to the potential promise of identifying targeted, contextually relevant control strategies. It also suggests the need for an inter-sectoral approach that combines education policy and health policy to reduce the burden of disease.

Mayala et al. [6] explore attitudes and knowledge about malaria and agriculture in farming households in Central Tanzania. They conducted a cross-sectional survey in a heavily agricultural area that experienced severe floods and crop losses in 2009–10. They conclude that despite very high ITN coverage (98.9%) and usage (96%) among the study population, the value of ITN may be reduced by local farming behaviors (such as rising early and remaining in fields overnight). Food insecurity in the community was reported to be widespread, and often coincided with the rainy season when both farming demands and malaria risk are comparatively high. Strong linkages between malaria, environment, and agriculture suggest the need for a policymaking approach which integrates health and agricultural strategies.

## Conclusion

At the global level, considerable progress has been made in reducing malaria through a mix of vector control and disease treatment interventions largely funded by international donors. To sustain this progress and achieve ambitious global goals, further ramp up of international donor funding will be required. In the future, control strategies will be more effective if a more targeted and contextually relevant approach is adopted that draws on expertise from multiple fields and sectors [7].

In addition, the value of integrating strategies into a more holistic, sustainable approach remains to be fully realized. This will require the concerted effort and commitment of decision-makers at multiple levels and sectors to pursue informed, evidence-based policymaking [8].

## Additional file

Additional file 1:
**Multilingual abstracts in the six official working languages of the United Nations.**

## Abbreviations

ITN: Insecticide treated net
ACT: Artemisinin-based combination therapies
