# Supplementary material for: Sustaining the gains made in malaria control and elimination
Source: Infect Dis Poverty. 2015 May 3;4:26. doi: 10.1186/s40249-015-0057-x (PMC4425886; doi:10.1186/s40249-015-0057-x)
Supplement: Additional file 1: — Multilingual abstracts in the six official working languages of the United Nations. [file 40249_2015_57_MOESM1_ESM.pdf]

Translation of the abstract into the six official working languages of the United Nations

إدامة المكاسب المحققة نتيجة مكافحة الملاريا والقضاء عليها

رندل أ. كرايمر وأدريان لسر

موجز

تمّ إحراز تقدم كبير خلال الـ25 عاماً الماضية في تخفيض عبء الملاريا، لكن ورغم ذلك، يبقى هناك تحدّيات عديدة لمواجهتها. نجمت هذه المكاسب عن استثمارات كبيرة في سلسلة من تدابير المكافحة التي استهدفت الملاريا. ويجد فانا والمؤلفون المشاركون أن ثمة علاقة قوية بين مستوى التعليم والاستعمال الصافي لتطفلن الدم لدى المرأة الحامل، ما يشير إلى الحاجة إلى وضع استراتيجيات مكافحة مستهدفة. كما أن ميالا والمولفين المشاركون يجدون أن ثمة صلات هامة بين الزراعة والملاريا ومقتضيات تعاون متعدد القطاعات من أجل مكافحة الملاريا.

Translated from English version into Arabic by Liliane Hatem, through

TRANSLATORS
WITHOUT BORDERS

从疟疾控制与消除的持续获益

兰德尔 A 克雷默， 阿德利恩 莱塞

摘要

近25年来，由于投入大量资金，疟疾控制措施在降低疟疾负担方面已取得重大进展，但仍存有很大的挑战。Fana和共同作者研究发现孕妇受教育水平和蚊帐使用情况与疟原虫感染有关，建议需实施相应的疟疾防控措施。Mayala和共同作者认为农业和疟疾关联紧密，疟疾防控需要跨部门协作。

Translated from English version into Chinese by Chen Jin, edited by Yang Pin, through

CDC
CHINESE CENTER FOR DISEASE CONTROL AND PREVENTION
NATIONAL INSTITUTE OF PARASITIC DISEASES

Maintenir les gains acquis dans la lutte contre le paludisme et son éradication

Randall A. Kramer et Adriane Lesser

Résumé

Même si des progrès importants ont été réalisés ces 25 dernières années dans la réduction de la charge du paludisme, des défis considérables subsistent. Ces progrès ont été possibles grâce à d’importants investissements effectués dans une série de mesures destinées à lutter contre le paludisme. Fana et des coauteurs mettent en évidence une forte corrélation entre le niveau d’éducation et l’utilisation du filet ainsi que la parasitémie du paludisme chez les femmes enceintes, d’où la nécessité d’établir des stratégies de lutte ciblées. Mayala et des collègues montrent qu’il existe des liens étroits entre l’agriculture et le paludisme, ce qui a des répercussions sur la collaboration intersectorielle dans la lutte contre le paludisme.

Translated from English version into French by Sabiha Azibi, through

TRANSLATORS
WITHOUT BORDERS

Поддержание результатов, достигнутых в искоренении и борьбе с малярией

Рэндалл А. Крамер и Адриан Лессер

Резюме

За последние 25 лет, достигнут серьезный прогресс в сокращении уровня заболеваемости малярией, однако основные задачи остаются нерешенными. Эти результаты были достигнуты при помощи крупных инвестиций в набор мер по

борьбе с малярией. Фана и соавторы обнаружили тесную связь между уровнем образования и использованием москитных сеток и наличием паразитов малярии в крови у беременных женщин, что предполагает необходимость адресных мер по борьбе с заболеванием. Маяла и коллеги обнаружили важную связь между сельским хозяйством и малярией с предпосылками для межсекторного сотрудничества с целью борьбы с малярией.

Translated from English version into Russian by Nurangiz Khodzharova, through

TRANSLATORS
WITHOUT BORDERS

Cómo mantener los logros alcanzados en el control y la eliminación de la malaria

Randall A. Kramer y Adriane Lesser

Resumen
En los últimos 25 años se ha logrado un progreso significativo en la reducción de la carga de la malaria. Sin embargo, todavía persisten desafíos importantes. Dichos logros son el resultado de grandes inversiones en una variedad de medidas de control dirigidas a la malaria. Fana y co-autores encontraron una sólida relación entre el nivel de educación y el uso de mosquiteros y la parasitemia en mujeres embarazadas con malaria, lo que sugiere que se necesitan estrategias de control dirigidas. Mayala y colegas encontraron vínculos importantes entre la agricultura y la malaria con implicancias para la colaboración intersectorial para el control de la malaria.

Translated from English version into Spanish by Maria Alejandra Aguada, through

TRANSLATORS
WITHOUT BORDERS
